# Detrended Multiple Cross-Correlation Coefficient applied to solar radiation, air temperature and relative humidity

**DOI:** 10.1038/s41598-019-56114-6

**Published:** 2019-12-24

**Authors:** Andrea de Almeida Brito, Heráclio Alves de Araújo, Gilney Figueira Zebende

**Affiliations:** 1 Federal Institute of Education, Science and Technology of Bahia, Paulo Afonso, Bahia Brazil; 2 SENAI CIMATEC, Salvador, Bahia Brazil; 3 National Institute of Meteorology, Brasília, Brazil; 4 State University of Feira de Santana, Bahia, Brazil

**Keywords:** Environmental impact, Statistics

## Abstract

Due to the importance of generating energy sustainably, with the Sun being a large solar power plant for the Earth, we study the cross-correlations between the main meteorological variables (global solar radiation, air temperature, and relative air humidity) from a global cross-correlation perspective to efficiently capture solar energy. This is done initially between pairs of these variables, with the Detrended Cross-Correlation Coefficient, *ρ*_*DCCA*_, and subsequently with the recently developed Multiple Detrended Cross-Correlation Coefficient, ${\boldsymbol{DM}}{{\boldsymbol{C}}}_{{\bf{x}}}^{{\bf{2}}}$. We use the hourly data from three meteorological stations of the Brazilian Institute of Meteorology located in the state of Bahia (Brazil). Initially, with the original data, we set up a color map for each variable to show the time dynamics. After, *ρ*_*DCCA*_ was calculated, thus obtaining a positive value between the global solar radiation and air temperature, and a negative value between the global solar radiation and air relative humidity, for all time scales. Finally, for the first time, was applied ${\boldsymbol{DM}}{{\boldsymbol{C}}}_{{\bf{x}}}^{{\bf{2}}}$ to analyze cross-correlations between three meteorological variables at the same time. On taking the global radiation as the dependent variable, and assuming that ${\boldsymbol{DM}}{{\boldsymbol{C}}}_{{\bf{x}}}^{{\bf{2}}}={\bf{1}}$ (which varies from 0 to 1) is the ideal value for the capture of solar energy, our analysis finds some patterns (differences) involving these meteorological stations with a high intensity of annual solar radiation.

## Introduction

Nowadays, we know of the requirement to seek sustainable energy sources in order to preserve the environment for future generations on Earth. As is known, the Sun with its radiation can be increasingly used in order to generate energy renewably and sustainably. There are essentially two main ways of harnessing solar energy: direct electricity generation and heating water in a boiler. From the point of view of electric energy, the two systems that are the most widespread are the heliothermic system (the radiation is converted first into thermal energy and then into electric energy) and the photovoltaic system (the radiation is converted directly into electric energy). But, realistically speaking, there are several ways of generating electricity. For example, according to the Brazilian Ministry of Mines and Energy^1^, the electrical matrix is made up of parts with the following percentages: Hydroelectric (63.7%), Thermal (27.2%), Wind (8.1%), and Solar (1.0%). We can see that most of the energy generation comes from hydroelectric plants, such as Itaipu (the world’s largest hydroelectric power plant). Of the 27% of non-renewable thermal sources, 8.1% come from natural gas, 9.1% biomass, 6.2% oil, 2.3% coal, 1.2% nuclear, and 0.2% from others types^1^.

Although hydroelectric plants use a renewable and a low-cost resource, they change the landscape, cause major deforestation, dam can break, as in Brumadinho^2^, and in general they cause damage to the fauna and the flora also, in many cases, families are displaced from their homes^3–6^.

Thus, there remains an incentive for research and development in this area for the furtherance of the policy of cleaner electricity generation, in order to replace non-renewable thermal energy by a cleaner renewable source such as solar radiation. Specifically, in Brazil the incentive for solar power energy is justified by the fact that the country has large areas with a high incidence of solar radiation; the semi-arid regions of the Northeast have the highest intensity of solar radiation over the year^7,8^. Presently, in the case of solar energy powered by photovoltaic cell panels, there is the disadvantage of their high initial cost and their low process efficiency, from 15% to 25%. Another aspect to be considered is the environmental impact caused by the use of the silicon in the production chain of the photovoltaic cells. However, the current and recurring challenge is to establish innovative manufacturing processes and use high-performance materials in order to obtain more efficiency in the collecting surface of the photovoltaic cells.

The efficiency of the photovoltaic cells depends not only on internal manufacturing factors, but also on external factors. External factors include shading by trees and/or clouds, rain, dust, solar radiation, air temperature, relative air humidity, and the wind speed and direction, among others. Since the external factors are not controllable, there has been research to understand the their effects. Due to its strong influence on the performance of photovoltaic cells, the solar radiation and the air temperature have been the most studied external factors^9–12^. There have also been many studies of the impact of the air temperature on photovoltaic cells, but there are still only a few studies that address the impact of these environmental factors in regions with a tropical climate, such as the Northeast of Brazil or that study how the variables are inter-related and interact^13^. Thus, not only should our attention be focused on energy generation: it is also important to have robust statistical tools in the area of the environmental sciences, with the purpose of analyzing how the external meteorological variables are related, as will be proposed in this paper with the DCCA multiple cross-correlation coefficient^14^.

## Data Set

**Figure 1:**
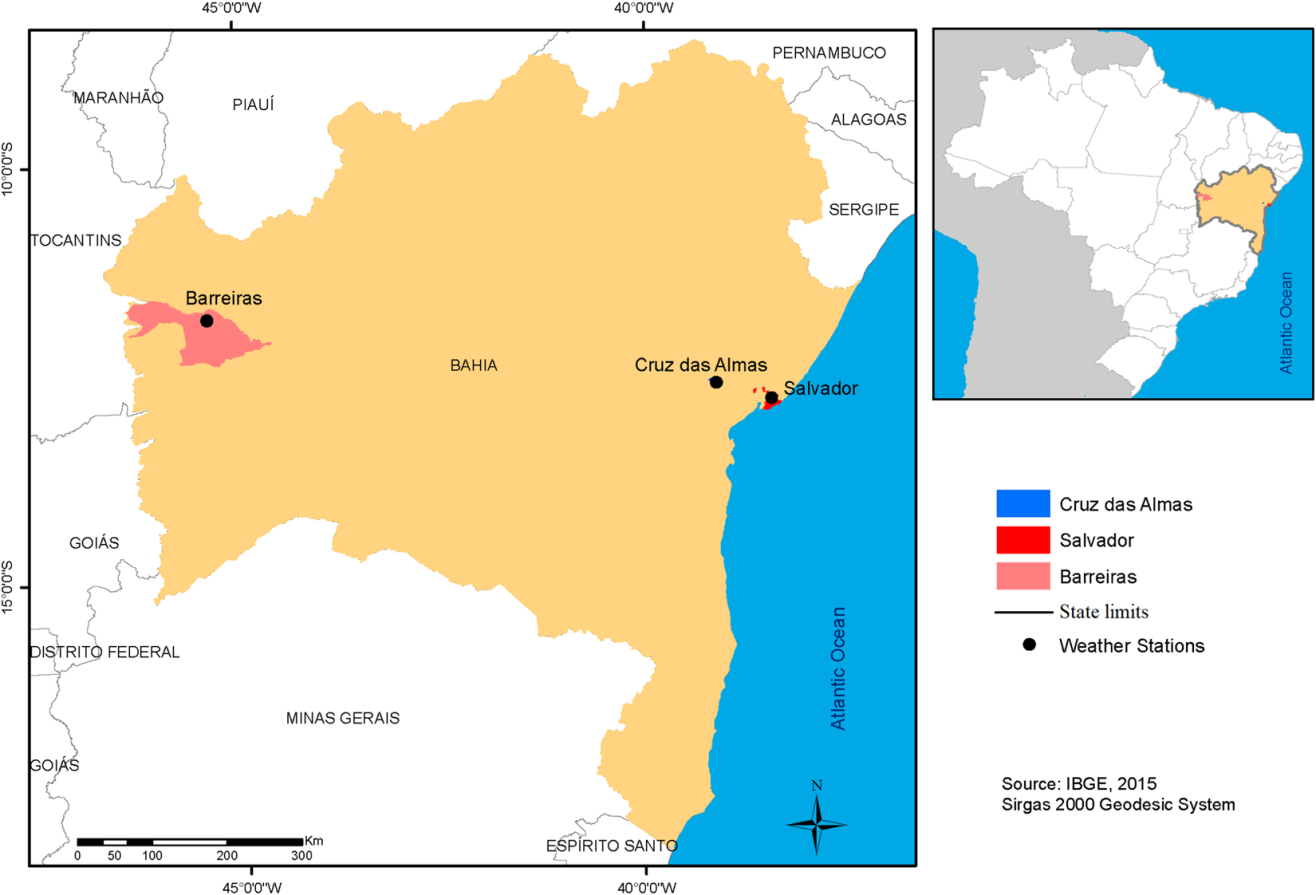
INMET automatic stations located in Bahia State (Brazil) at the cities of: Barreiras (Elev: 474 m), Cruz das Almas (Elev: 220 m), and Salvador (Elev: 48 m).

Taking into consideration that the global solar radiations is measured while we have sunlight on the sensor (pyranometer), hence the data was taken hourly from 10 to 21 h UTC (Coordinated Universal Time). Our data were obtained from meteorological stations managed by Brazilian Institute of Meteorology (INMET)^15^. Therefore, in order to study its potential for solar power we chose three meteorological stations localized in the state of Bahia (Brazil), see Fig. 1. These stations are important because they are the ones that have the best databases (about global solar radiation)^15^, and because they have the following characteristics, see Table 1.

**Table 1:** Station Info:

Station	INMET code^15^	Start	End	*N* points
Barreiras	A402	12/22/2001	12/11/2018	62328
Cruz das Almas	A406	10/04/2005	12/11/2018	48084
Salvador	A401	10/06/2000	12/11/2018	63264

Barreiras the most populous and important agricultural center in the western region, is an important producer of cotton and soybeans. Cruz das Almas important center in the *Recôncavo*, region with some agricultural research centers such as EMBRAPA. Salvador the capital and economic center of Bahia with more than 2.9 million inhabitants.

**Figure 2:**
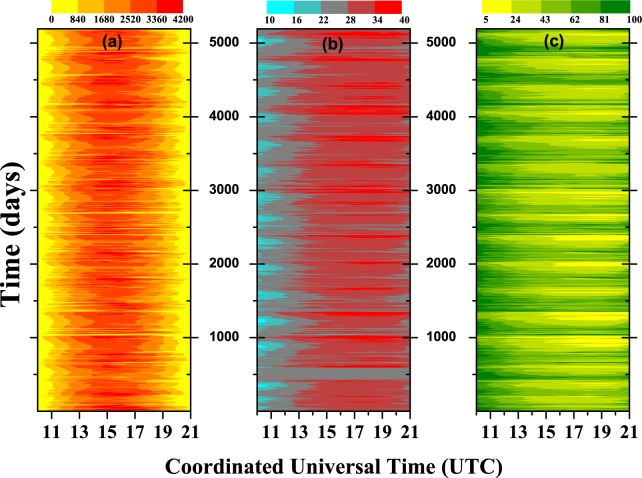
Color map for Barreiras station with: (**a**) global solar radiation (KJ/m^2^), (**b**) air temperature (°C), and (**c**) relative air humidity (%).

**Figure 3:**
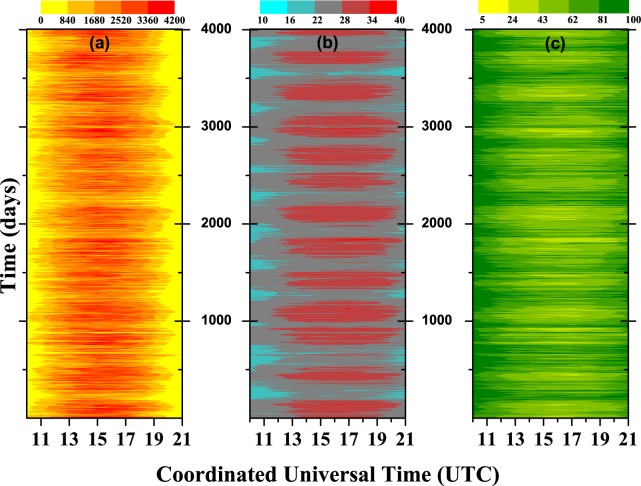
Color map for Cruz das Almas station with: (**a)** global solar radiation (KJ/m^2^), (**b**) air temperature (°C), and (**c**) relative air humidity (%).

**Figure 4:**
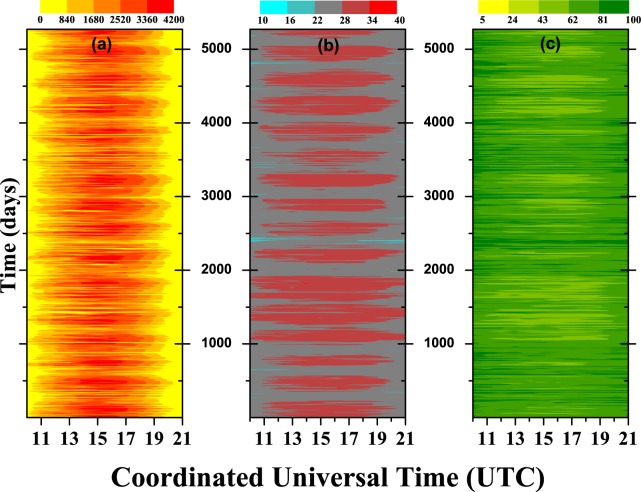
Color map for Salvador station with: (**a**) global solar radiation (KJ/m^2^), (**b**) air temperature (°C), and (**c**) relative air humidity (%).
- **BAR**: 0 ≤ Rad. ≤ 4051 (KJ/*m*^2^)$\therefore$11 ≤ Temp. ≤ 40 (°C)$\therefore$8 ≤ Hum. ≤ 100 (%);
- **CRUZ**: 0 ≤ Rad. ≤ 4148 (KJ/*m*^2^)$\therefore$14 ≤ Temp. ≤ 38 (°C)$\therefore$15 ≤ Hum. ≤ 100 (%);
- **SSA**: 0 ≤ Rad. ≤ 4088 (KJ/*m*^2^)$\therefore$19 ≤ Temp. ≤ 35 (°C)$\therefore$33 ≤ Hum. ≤ 100 (%).

Our first choice of analysis was to temporally order the data in a color map, with the three meteorological variables side by side, which are:

- (a) Global Solar Radiation (KJ/m^2^);
- (b) Air Temperature (°C);
- (c) Relative Air Humidity (%).
  For each station, these variables can be see in Fig. 2 (Barreiras), Fig. 3 (Cruz da Almas), and Fig. 4 (Salvador). In these figures, a day (with 12 h) start at 10 h and ends at 21 h (UTC). It can be seen that the maximum global solar radiation is usually concentrated around the peak of sunlight ($\simeq$15 h). Logically, there are fluctuations in this value, depending on the season of the year or the time of day. We can also note in the color map a direct relation between the variables, but the color map is not able to robustly quantify that value; it gives us a beautiful visual display of information as to the dynamic changes in the variables. The next step is to quantitatively describe the features of the data for the whole of the period: for example, if we take the minimum and maximum values one can see:

But, these statistics can be more refined if the seasons are considered. More details about these statistics can be seen in the results section below.

## Results

### Descriptive statistical

**Figure 5:**
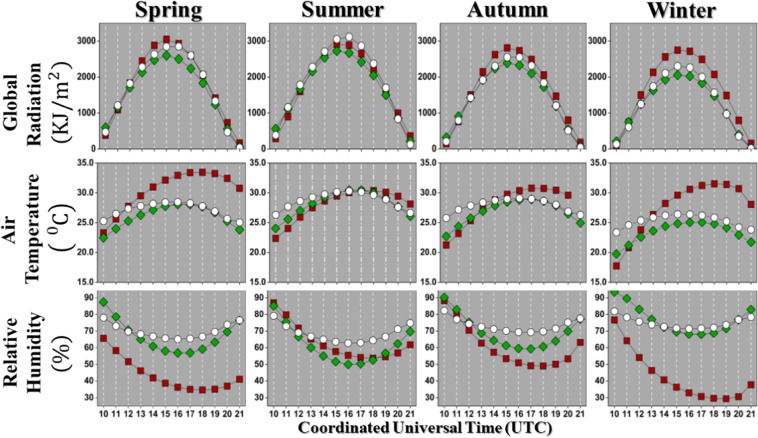
Mean values for global solar radiation, air temperature, and relative air humidity measured at every hour (UTC) for the four seasons (Spring, Summer, Autumn, Winter). The results are displayed for Barreiras (), Cruz das Almas (), and Salvador (○) stations.

Initially as the results, we compute the mean values in the point of view of the annual seasons in the southern hemisphere. In this sense to simplify the climatological calculations and keep them uniform we choose the meteorological definition for seasons^16^ with:

- **Spring** from September/01 to November/30;
- **Summer** from December/01 to February/28;
- **Autumn** from March/01 to May/31;
- **Winter** from June/01 to August/31.
  Figure 5 present these mean values (performed at every time (UTC)) for the global solar radiation, air temperature, and relative air humidity at each season (Spring, Summer, Autumn, Winter). It is possible to observe that the radiation in the sensor has a distribution (apparently normal) characterized by a mode varying with the season. The value for maximum solar global radiation is around 15 h (UTC) and this intensity depends on the location (see Table 2).

**Table 2:** Mean of global solar radiation with maximum values.

	Time (UTC)	Max. Rad. (KJ/m^2^)	Temp. (°C)	Hum. (%)	
BAR	15	3048	32	39	Spring
	15	2914	29	58	Summer
	15	2808	29	53	Autumn
	15	2747	30	36	Winter
CRUZ	15	2595	28	58	Spring
	15	2722	30	52	Summer
	15	2388	28	61	Autumn
	15	2055	25	70	Winter
SSA	16	2849	28	65	Spring
	16	3115	30	63	Summer
	15	2550	29	70	Autumn
	15	2299	26	72	Winter

First column shows the time and subsequent columns shows air temperature and relative air humidity.
  The maximum global solar radiation is at 15 h, except at Salvador in the spring and summer, that is at 16 h. The peak of maximum air temperature and minimum relative air humidity has a greater variation depending on the annual season. There is a clear inverse relation between air temperature and relative air humidity, more evidenced for Barreiras station, as shown in^17^. But, depending on the location (northern hemisphere) such a relation is not always true^18^. In this paper we also measured other moments, that are:
- Standard deviation (%): $$sd( \% )=\frac{sd\,\ast \,100}{\langle x\rangle }$$
- Skewness: $$sk\equiv \frac{n}{(n-1)\,(n-2)}\,{\sum }_{i=1}^{n}\,{(\frac{{x}_{i}-\langle x\rangle }{sd})}^{3}$$
- Kurtosis: $${K}_{e}\equiv \frac{\frac{1}{n}\,{\sum }_{i=1}^{n}\,{({x}_{i}-\langle x\rangle )}^{4}}{{[\frac{1}{n}{\sum }_{i=1}^{n}{({x}_{i}-\langle x\rangle )}^{2}]}^{2}}-3$$

in this case, $\langle x\rangle$ is the mean and *sd* is the sample standard deviation, see Table 3 with the results. We can see that the highest relative standard deviation is for the global solar radiation (≈35%), except in the Spring and Winter for Barreiras station, where the relative air humidity has the highest. For the skewness, we note in general values different from 0 (but close), indicating that our data-set diverges a little from the mean with positive or negative values. There is an excess Kurtosis, ${K}_{e}\ne 0$ for most of the values found in the Table 3, with ${K}_{e} < 0$ (platykurtic distribution) and with ${K}_{e} > 0$ (leptokurtic distribution). These results indicate that the meteorological time series is non-stationary. But, these descriptive statistics are well known and we want to propose something innovative in the study of direct or indirect relations between the main meteorological variables. With ${\rho }_{{{\rm{x}}}_{i},{{\rm{x}}}_{j}}$ and $DM{C}_{{\rm{x}}}^{2}$, we will succeed.

**Table 3:** Descriptive statistics of the variables with: Standard deviation (%), Skewness, and Kurtosis.

		Spring			Summer			Autumn			Winter		
		Rad.	Tem.	Hum.	Rad.	Tem.	Hum.	Rad.	Tem.	Hum.	Rad.	Tem.	Hum.
**B**	*sd*(%)	33.1	10.1	44.3	40.3	9.3	23.1	33.3	8.0	21.9	18.3	7.8	21.7
**A**	*sk*	−1.0	−0.8	0.9	−0.6	−0.4	0.2	−0.6	−0.7	0.4	−1.5	−0.3	0.7
**R**	*K*_*e*_	1.0	0.7	0.1	−0.4	0.1	0.0	0.7	0.6	0.1	5.4	0.5	1.9
**C**	*sd*(%)	37.7	8.6	16.4	32.4	5.8	16.6	42.5	9.3	19.1	43.9	6.5	12.1
**U**	*sk*	0.3	−0.5	0.5	−0.4	−0.8	0.8	0.0	−0.2	−0.1	1.0	−0.2	0.0
**Z**	*K*_*e*_	8.2	0.0	0.1	0.1	1.5	1.0	−0.3	−0.3	−0.4	18.8	0.2	−0.1
**S**	*sd*(%)	35.8	6.7	12.0	33.0	5.6	12.0	46.6	7.0	12.6	39.0	6.0	12.0
**S**	*sk*	−0.6	−0.5	0.5	−0.8	−0.6	0.6	−0.1	−0.4	0.3	0.0	−0.3	0.0
**A**	*K*_*e*_	0.2	0.4	0.6	0.5	1.1	0.6	0.3	−0.2	−0.5	0.8	0.1	0.0

### DCCA cross-correlation coefficient ${{\boldsymbol{\rho }}}_{{{\bf{x}}}_{{\boldsymbol{i}}},{{\bf{x}}}_{{\boldsymbol{j}}}}$

**Figure 6:**
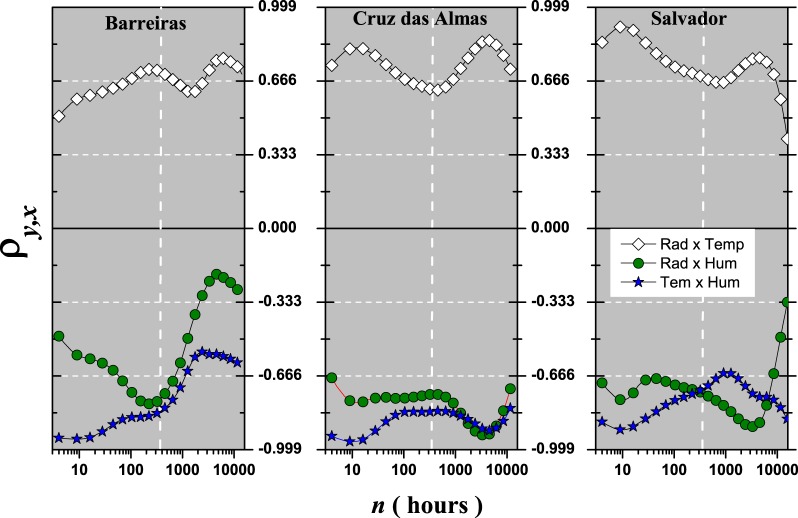
${\rho }_{{{\rm{x}}}_{i},{{\rm{x}}}_{j}}$ as a function of *n* for all stations and combinations: Solar Radiation × Air Temperature (◇), Solar Radiation × Relative Humidity (), and Air Temperature × Relative Humidity (). Vertical lines show $n=360$ (30 days).

Figure 6 presents the values of ${\rho }_{{{\rm{x}}}_{i},{{\rm{x}}}_{j}}$ for the cross-correlations between global solar radiation × air temperature, global solar radiation × relative air humidity, and air temperature × relative air humidity (these are particular cases of $DM{C}_{{\rm{x}}}^{2}$ for a pair of time series). We can see clearly that the variables are related for all time scales *n* and for all meteorological stations, because ${\rho }_{{{\rm{x}}}_{i},{{\rm{x}}}_{j}}\ne 0$. Thus, due to the importance of the air temperature as a meteorological variable to solar power energy, Fig. 6 (◇) shows ${\rho }_{{{\rm{x}}}_{i},{{\rm{x}}}_{j}}$ between global solar radiation and air temperature. In this case ${\rho }_{{{\rm{x}}}_{i},{{\rm{x}}}_{j}} > 0$, with a strong (mostly) DCCA cross-correlation for all time scales *n* and meteorological stations. Specifically, for $n < 360$ (30 days), Fig. 6 (◇) shows that ${\rho }_{{{\rm{x}}}_{i},{{\rm{x}}}_{j}}$ is lower for Barreiras than for with Cruz das Almas and Salvador at the same time scale. For $n=360$, ${\rho }_{{{\rm{x}}}_{i},{{\rm{x}}}_{j}}$ has approximately the same value of 0.666 for these three stations. But, for $n > 360$, there is a maximum value for ${\rho }_{{{\rm{x}}}_{i},{{\rm{x}}}_{j}}$ around $n\approx 4400$ (≈365 days); this probably shows the cyclical annual effect. Analyzing the DCCA cross-correlation between the global solar radiation and the relative air humidity in Fig. 6 () it is possible to observe that ${\rho }_{{{\rm{x}}}_{i},{{\rm{x}}}_{j}} < 0$ for all time scales *n* and stations. Again, if we take as reference $n=360$, patterns for each of the stations can be identified. For the DCCA cross-correlation between air temperature and relative air humidity, Fig. 6 (), ${\rho }_{{{\rm{x}}}_{i},{{\rm{x}}}_{j}} < 0$ which agrees with^17^.

But, if we want to study a cross-correlation between three (global solar radiation, air temperature and relative air humidity) or more variables, we must apply a method that generalizes ${\rho }_{{{\rm{x}}}_{i},{{\rm{x}}}_{j}}$, such as $DM{C}_{{\rm{x}}}^{2}$. The results of its application will be presented in the following.

### DCCA multiple cross-correlation coefficient ${\boldsymbol{DM}}{{\boldsymbol{C}}}_{{\bf{x}}}^{{\bf{2}}}$

**Figure 7:**
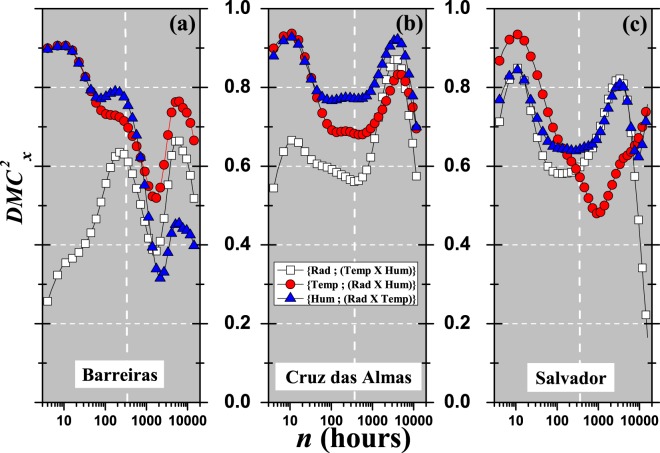
$DM{C}_{{\rm{x}}}^{2}$ as a function of *n* between {Global Radiation; (Air Temperature × Relative Humidity)} (□), {Air Temperature; (Global Radiation × Relative Humidity)} (), and {Relative Humidity; (Global Radiation × Air Temperature)} () for all stations: (**a**) Barreiras, (**b**) Cruz das Almas, and (**c**) Salvador. Vertical line show $n=360$ (30 days).

Figure 7 presents $DM{C}_{{\rm{x}}}^{2}(n)$ for global solar radiation, air temperature and relative air humidity at the same time. From this figure, it can be seen that the variables are related globally, because they have DCCA multiple cross-correlations that range from weak to very strong. In an intuitive way, as introduced here, this result depends on the dependent variable {*y*}, the station, and the time scale involved. Figure 7 show that $DM{C}_{{\rm{x}}}^{2}$ for {Air Temperature; (Global Radiation × Relative Humidity)} () behave similarly, for $n < 360$, for all stations, but differ for $n > 360$. Up to the value $n\simeq 70$ {Air Temperature; (Global Radiation × Relative Humidity)} and {Relative Humidity; (Global Radiation × Air Temperature)} yield approximately the same value of $DM{C}_{{\rm{x}}}^{2}$, going from very strong to strong DCCA multiple cross-correlation. For the Salvador station, $DM{C}_{{\rm{x}}}^{2}$ (□) are closer to ( and ) than they are for the Barreiras station, mainly for small time scales *n*.

**Figure 8:**
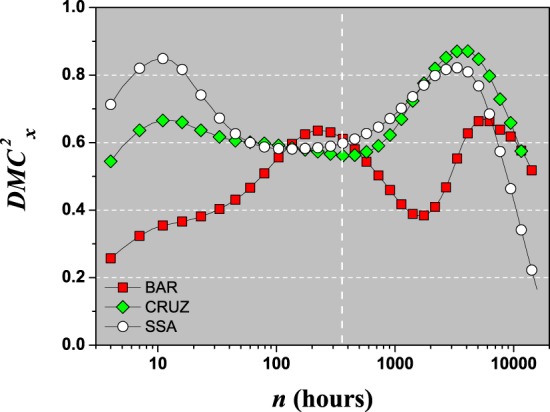
$DM{C}_{{\rm{x}}}^{2}$ as a function of *n* between {Global Radiation; (Air Temperature × Relative Humidity)} for the Barreiras (), Cruz das Almas (), and Salvador (○) stations. Vertical line show $n=360$ (30 days).

Our goal here is to study how the meteorological variables are related. We believe that this can help promote the efficiency of capturing solar energy in photovoltaic cells located in a certain region with climatic variations. To this end, looking at $DM{C}_{{\rm{x}}}^{2}(n)$ and taking as a dependent variable the global solar radiation, we have the following results, Fig. 8. Setting $DM{C}_{{\rm{x}}}^{2}=1$ for maximum efficiency in capturing solar energy for all time scales, both in short and long-term, we can analyze separately each location and observe the dependence of $DM{C}_{{\rm{x}}}^{2}$ on the time scale and location and define in this way the potential efficiency of each location. In our case study, all stations have $DM{C}_{{\rm{x}}}^{2} > 0.2$ with levels of the multiple cross-correlation lying between weak and very strong (see Table 4). For small time scales, $n < 10$, we have, in decreasing order of their multiple cross-correlation, Salvador (○), Cruz das Almas (), and Barreiras (). At $n\simeq 360$ (30 days), all stations have approximately the same value, $DM{C}_{{\rm{x}}}^{2}\simeq 0.6$. For long time scales, there is a peak of $DM{C}_{{\rm{x}}}^{2}$ for Salvador and Barreiras at $n\approx 3652$ (one year), of approximately 0.8, whereas for Barreiras this value is below 0.6.

**Table 4:** DCCA multiple cross-correlation coefficient levels.

Condition	${\boldsymbol{DM}}{{\boldsymbol{C}}}_{{\bf{x}}}^{{\bf{2}}}$
very-weak	$0.0\mapsto 0.2$
weak	$0.2\mapsto 0.4$
medium	$0.4\mapsto 0.6$
strong	$0.6\mapsto 0.8$
very-strong	$0.8\mapsto 1.0$

## Discussion

Taking into account the global solar radiation, the air temperature and the relative air humidity, we have studied the cross-correlations from a global perspective, by using the multiple detrended cross-correlation coefficient, $DM{C}_{{\rm{x}}}^{2}$. Initially we provide, for better data visualization, the descriptive statistics of these time series. Then, with their mean values and with the capture and or generation of solar energy in view, we found the maximum global solar radiation value. It can be seen here that usually this maximum is concentrated at 15 h (UTC), but with different air temperature and relative air humidity depending on the season. Logically, with standard deviation, skewness, and kurtosis, we can infer whether or not the probability distribution function approach the normal distribution. In this paper, it can be seen that depending on the season, the distributions deviate from the normal, characterizing these time series as non-stationary. But, this classical statistical analysis only takes into consideration each variable separately. To analyze the relations between them in pairs (or more) we must apply a statistical tool that has this capability.

Developed by Zebende^19^, Detrended Cross-correlation Coefficient, ${\rho }_{{{\rm{x}}}_{i},{{\rm{x}}}_{j}}$, was constructed in order to analyze the cross-correlations between pairs of non-stationary time series. It is robust if compared, for example, with the Pearson’s coefficient^20^. In this sense, the values of ${\rho }_{{{\rm{x}}}_{i},{{\rm{x}}}_{j}}$ between the global solar radiation, air temperature, and relative air humidity (in pairs) depend on the geographical location and the time scale, for these three meteorological stations chosen here. There is a positive relation between global solar radiation and air temperature and an inverse relation between global solar radiation and relative air humidity.

As mentioned in the introduction, the efficiency of a photovoltaic cell depends mainly on internal factors, but that the external factors are also important, as well as their inter-relations. Our goal here was to apply the multiple DCCA cross-correlation, $DM{C}_{{\rm{x}}}^{2}$, to study globally the relation between three (main) variables involved in solar energy. It is noteworthy that such application has not yet been performed and this paper is the pioneer to treat together these three variables at the same time with $DM{C}_{{\rm{x}}}^{2}$. Thus, assuming $DM{C}_{{\rm{x}}}^{2}=1$ to be the ideal value for solar energy capture, if we have global solar radiation as the dependent variable, than for the meteorological stations of Barreiras, Cruz das Almas, and Salvador, located in the northeast of Brazil, an area with a high intensity of annual solar radiation, we did our analysis and noticed some patterns (differences). Because $DM{C}_{{\rm{x}}}^{2}$ is a function of the time scale *n*, we can determine this multiple coefficient for small, medium, and long time scales. For example, for $n=12$ (small time scale), the Salvador station has the best value for capture solar energy according to $DM{C}_{{\rm{x}}}^{2}$, with very-strong value. For $n=360$ (one month) all stations have an intermediate value for $DM{C}_{{\rm{x}}}^{2}$, that is, between medium and strong. But, for long time scales, Barreiras and Cruz das Almas have the best values for capture solar energy if compared to Salvador.

Finally, it is worth pointing out that in a certain way the stations are close to each other, and that a study for other stations around the planet would be very welcome. But, the purpose of this paper was to apply a new method to the analysis of multiple cross-correlations between meteorological variables in a global (innovative) way. In conclusion, as the expression for multiple correlation is quite general, other variables can be employed, such as atmospheric pressure and wind speed, among others, adding even more information to the calculation of $DM{C}_{{\rm{x}}}^{2}$.

## Methods

For DCCA multiple cross-correlation coefficient^14^ presentation, we employ the DCCA cross-correlation coefficient ${\rho }_{{{\rm{x}}}_{i},{{\rm{x}}}_{j}}$^19^, which is defined in terms of the ${F}_{DFA}(n)$^21^ and the ${F}_{DCCA}^{2}(n)$ fluctuation function^22^:

1 $${\rho }_{{{\rm{x}}}_{i},{{\rm{x}}}_{j}}(n)\equiv \frac{{F}_{DCCA}^{2}(n)}{{F}_{DF{A}_{\{{{\rm{x}}}_{i}\}}(n)}\,{F}_{DF{A}_{\{{{\rm{x}}}_{j}\}}(n)}}.$$

The DCCA cross-correlation coefficient in Eq. 1 ranges between −$1\le {\rho }_{{{\rm{x}}}_{i},{{\rm{x}}}_{j}}\le 1$, and has been applied in several papers, such as^20,23–27^, among many others^28^. It is possible to generalize the idea behind ${\rho }_{{{\rm{x}}}_{i},{{\rm{x}}}_{j}}$ to more than two variables, and such a new multiple coefficient is referred to as the DCCA multiple cross-correlation coefficient, denoted by $DM{C}_{{\rm{x}}}^{2}$^14^:

2 $$DM{C}_{{\rm{x}}}^{2}(n)={\Phi }_{{\rm{y}},{\rm{x}}}{(n)}^{T}{\rho }^{-1}(n){\Phi }_{{\rm{y}},{\rm{x}}}(n)$$

where,

3 $${\Phi }_{{\rm{y}},{\rm{x}}}{(n)}^{T}\equiv [{\rho }_{{\rm{y}},{{\rm{x}}}_{1}}(n),{\rho }_{{\rm{y}},{{\rm{x}}}_{2}}(n),\ldots ,{\rho }_{{\rm{y}},{{\rm{x}}}_{i}}(n)]$$

is the detrended cross-correlations vector between the independent variables (*x*_*i*_) and the dependent variable (*y*), and ${\rho }^{-1}(n)$ is the matrix inverse of the detrended cross-correlation matrix of the independent variables, defined as

4 $${\rho }^{-1}(n)\equiv {(\begin{array}{ccccc}1 & {\rho }_{{\text{x}}_{1},{\text{x}}_{2}}(n) & {\rho }_{{\text{x}}_{1},{\text{x}}_{3}}(n) & \cdots  & {\rho }_{{\text{x}}_{1},{\text{x}}_{i}}(n)\\ {\rho }_{{\text{x}}_{1},{\text{x}}_{2}}(n) & 1 & {\rho }_{{\text{x}}_{2},{\text{x}}_{3}}(n) & \cdots  & {\rho }_{{\text{x}}_{2},{\text{x}}_{i}}(n)\\ : & : & : & \cdots  & :\\ {\rho }_{{\text{x}}_{1},{\text{x}}_{i}}(n) & {\rho }_{{\text{x}}_{2},{\text{x}}_{i}}(n) & {\rho }_{{\text{x}}_{3},{\text{x}}_{i}}(n) & \cdots  & 1\end{array})}^{-1}$$

This matrix is symmetric because ${\rho }_{{{\rm{x}}}_{i},{{\rm{x}}}_{j}}(n)={\rho }_{{{\rm{x}}}_{j},{{\rm{x}}}_{i}}(n)$, ${\rho }_{{{\rm{x}}}_{i},{{\rm{x}}}_{i}}(n)=1$, and $0\le DM{C}_{{\rm{x}}}^{2}(n)\le 1$, see Table 4 for more details about the levels. For example, if the global solar radiation is {*y*}, the air temperature is {*x*_1_}, and the relative air humidity is {*x*_2_}, then

5 $$DM{C}_{{\rm{x}}}^{2}(n)=\frac{{\rho }_{{\rm{y}},{{\rm{x}}}_{1}}^{2}(n)+{\rho }_{{\rm{y}},{{\rm{x}}}_{2}}^{2}(n)-2{\rho }_{{\rm{y}},{{\rm{x}}}_{1}}(n){\rho }_{{\rm{y}},{{\rm{x}}}_{2}}(n){\rho }_{{{\rm{x}}}_{1},{{\rm{x}}}_{2}}(n)}{1-{\rho }_{{{\rm{x}}}_{1},{{\rm{x}}}_{2}}^{2}(n)}$$
